# Placental multimodal MRI prior to spontaneous preterm birth <32 weeks' gestation: An observational study

**DOI:** 10.1111/1471-0528.17901

**Published:** 2024-07-02

**Authors:** Megan Hall, Natalie Suff, Paddy Slator, Mary Rutherford, Andrew Shennan, Jana Hutter, Lisa Story

**Affiliations:** ^1^ Centre for the Developing Brain, St Thomas' Hospital King's College London London UK; ^2^ Department of Women and Children's Health, St Thomas' Hospital King's College London London UK; ^3^ Cardiff University Brain Research Imaging Centre Cardiff University Cardiff UK; ^4^ School of Computer Science and Informatics Cardiff University Cardiff UK; ^5^ Smart Imaging Lab, Radiological Institute University Hospital Erlangen Erlangen Germany

**Keywords:** chorioamnionitis, diffusion imaging, placental MRI, PPROM, preterm birth

## Abstract

**Objective:**

To utilise combined diffusion‐relaxation MRI techniques to interrogate antenatal changes in the placenta prior to extreme preterm birth among both women with PPROM and membranes intact, and compare this to a control group who subsequently delivered at term.

**Design:**

Observational study.

**Setting:**

Tertiary Obstetric Unit, London, UK.

**Population:**

Cases: pregnant women who subsequently spontaneously delivered a singleton pregnancy prior to 32 weeks' gestation without any other obstetric complications. Controls: pregnant women who delivered an uncomplicated pregnancy at term.

**Methods:**

All women consented to an MRI examination. A combined diffusion‐relaxation MRI of the placenta was undertaken and analysed using fractional anisotropy, a combined T2*‐apparent diffusion coefficient model and a combined T2*‐intravoxel incoherent motion model, in order to provide a detailed placental phenotype associated with preterm birth. Subgroup analyses based on whether women in the case group had PPROM or intact membranes at time of scan, and on latency to delivery were performed.

**Main Outcome Measures:**

Fractional anisotropy, apparent diffusion coefficients and T2* placental values, from two models including a combined T2*‐IVIM model separating fast‐ and slow‐flowing (perfusing and diffusing) compartments.

**Results:**

This study included 23 women who delivered preterm and 52 women who delivered at term. Placental T2* was lower in the T2*‐apparent diffusion coefficient model (*p* < 0.001) and in the fast‐ and slow‐flowing compartments (*p* = 0.001 and *p* < 0.001) of the T2*‐IVIM model. This reached a higher level of significance in the preterm prelabour rupture of the membranes group than in the membranes intact group. There was a reduced perfusion fraction among the cases with impending delivery.

**Conclusions:**

Placental diffusion‐relaxation reveals significant changes in the placenta prior to preterm birth with greater effect noted in cases of preterm prelabour rupture of the membranes. Application of this technique may allow clinically valuable interrogation of histopathological changes before preterm birth. In turn, this could facilitate more accurate antenatal prediction of preterm chorioamnionitis and so aid decisions around the safest time of delivery. Furthermore, this technique provides a research tool to improve understanding of the pathological mechanisms associated with preterm birth in vivo.

## 1 INTRODUCTION

Spontaneous preterm birth complicates up to 10% of deliveries annually and is the leading cause of mortality in the under‐5 year's age group, with risk inversely associated with gestational age at delivery.^1^ Chorioamnionitis with or without funisitis is a common pathogenesis of preterm labour, particularly at extreme gestation: while the rate of chorioamnionitis in preterm delivery is between 40% and 70% overall,^2^ this is as high as 94% in deliveries prior to 24 weeks' gestation.^3^ However, other placental pathologies, particularly maternal vascular malperfusion lesions, have been implicated in the aetiology of preterm birth independent of infection.^4^

A placental diagnosis can only be made histopathologically after delivery. In the case of chorioamnionitis, there are no non‐invasive antenatal investigations with sufficient sensitivity and specificity for reliable use clinically.^5^ While maternal pyrexia of ≥38.0°C has good sensitivity in high‐risk women (95%–100%), this is a late finding in the disease process and earlier changes such as offensive discharge or fetal tachycardia have poor sensitivity in disease predicition (5%–22% and 40%–70%, respectively).^2^ Although amniocentesis is of some value in predicting infection, the invasive nature of the test and its association with further increasing the risk of preterm birth means it is not universally acceptable to patients or clinicians.^6^ This is of particular concern given that, as well as being a cause of maternal sepsis and mortality,^7^ neonates born preterm following chorioamnionitis are known to have more severe outcomes than gestation‐matched controls, including higher rates of cerebral palsy.^8^, ^9^

The complex histopathological changes associated with chorioamnionitis, such as increased neutrophil invasion, increased cytokine concentrations and fibrin deposition lend themselves well to assessment via multimodal functional MRI, so raising the potential that this could be a tool for antenatal assessment of the placenta.^10^ Diffusion techniques can interrogate tissue microstructure, and T2* relaxometry is utilised as a proxy assessment of tissue oxygenation. Both techniques allow for visualisation and quantification of tissue properties spatially on parameter maps and can produce averaged region‐specific mean values.

As well as capturing normal changes over gestation,^11^ T2* has been demonstrated to be significantly lower in women with pre‐eclampsia and fetal growth restriction.^12^, ^13^ Diffusion MRI allows for further interrogation of normal change across gestation,^14^ and is altered in cases of maternal vascular malperfusion.^15^ Advances in combined T2*‐diffusion acquisition techniques, giving access to a larger parameter space and hence a wider range of properties and analysis techniques, have shown novel insights,^16^ including reduced T2* primarily from the perfusing compartment in women with preterm prelabour rupture of the membranes (PPROM)—a finding hypothesised to relate to recruitment of neutrophils and cytokines to the region.^17^

This all supports the use of advanced MRI techniques to match the complexity of placental function. This study aims to utilise diffusion‐relaxation techniques to interrogate antenatal changes in the placenta prior to extreme preterm birth among both women with PPROM and intact fetal membranes, and compare findings to a control group who subsequently delivered at term.

## 2 METHODS

### 2.1 Patient participation

Women with experience of preterm birth were consulted prior to the development of this study. They continue to be involved in all stages of the wider body of work, of which this is a part, via the Guy's and St Thomas' NHS Foundation Trust Preterm Birth PPI Group and the patient support and advocacy group Little Heartbeats.

### 2.2 Recruitment of participants

Women at high risk of preterm birth prior to 32 weeks' gestation were prospectively recruited from a teaching hospital in London. Inclusion criteria were PPROM, exposed membranes or a combined QUIPP risk assessment of >75% chance of delivery prior to 32 weeks.^18^ A control group of low‐risk women who subsequently delivered an uncomplicated singleton pregnancy at term without any maternal or fetal complications were also included. Exclusion criteria included active labour, inability to give informed consent, metallic implant contraindicating MRI, other obstetric or fetal conditions, and multiple pregnancies. Any woman who developed an obstetric pathology demonstrated to have an impact on placental imaging or histopathology later in her pregnancy was excluded.

### 2.3 Image acquisition

Scanning was undertaken in supine position on a 3T Philips Achieva scanner (Philips, Best, Netherlands) using a 32‐channel cardiac coil. Continuous heart rate and peripheral oxygen saturation monitoring, and intermittent blood pressure monitoring was undertaken during the scan. Total scan time was around 60 min with a break after 30 min as anatomical and functional data not described here were also acquired.

Combined T2*‐diffusion scanning using a multi‐echo gradient echo diffusion‐weighted single‐shot echo planar imaging sequence was then undertaken coronal to the mother using parameters optimised for placental MRI.^19^ Diffusion preparation parameters were as follows: three gradient directions at all of *b* = [5, 10, 25, 50, 100, 200, 400, 600, 1200 and 1600] s/mm^2^; eight directions at *b* = 18 s/mm^2^; seven directions at *b* = 36 s/mm^2^; and 15 directions at *b* = 800 s/mm^2^. Each diffusion preparation was obtained at four echo times (TE; 78, 114, 150 and 186 ms). Total acquisition time for the sequence was under 9 min.^20^

### 2.4 Image analysis

Diffusion‐relaxation data were motion corrected and reconstructed using a previously described technique (all motion correction and reconstruction by JH, 16 years placental MRI experience).^17^, ^19^, ^21^ Manual segmentation of the placental parenchyma was undertaken on ITK‐SNAP^22^ using a conservative segmentation of the placental parenchyma in order to exclude surrounding tissue (segmented by MH, 3 years placental MRI experience. Interclass correlation coefficient with LS, 16 years placental MRI experience 0.99) and thresholding of T2* data to exclude supraphysiological values of >200 ms. Fractional anisotropy, a diffusion parameter was obtained from the diffusion data at the first TE.^23^ Subsequent analyses were undertaken using in‐house python scripts and extensions to the diffusion microstructure imaging in python library for diffusion models.^24^, ^25^ Two combined diffusion‐relaxometry models were fit to the data: a simple T2*‐ADC model (Equation 1) and a two‐compartment T2*‐IVIM model that differentiates fast and slow diffusion, taken to represent perfusing and diffusing placental blood respectively (Equation 2):

(1) $$S\left(\mathrm{TE},b\right)=S_{0}e^{-\left(\mathrm{TE}-{\mathrm{TE}}_{\min}\right)/T2*e^{-b\mathrm{ADC}}}$$

Where TE = echo time; TE_min_ = shortest echo time acquired; *S*_0_ = signal at the shortest echo time with diffusion weighting of zero; *b* = *b*‐value; ADC = apparent diffusion coefficient.

(2) $$S\left(\mathrm{TE},b\right)=S_{0}\;\left[{\mathrm{fe}}^{-\left(\mathrm{TE}-{\mathrm{TE}}_{\min}\right)/{T2}_{\text{fast}}^{*}e-\mathrm{bD}*+\left(1-f\right)e^{-\left(\mathrm{TE}-{\mathrm{TE}}_{\min}\right)/{T2}_{\text{slow}}^{*}e-b\mathrm{ADC}}}\right]$$

Where TE = echo time; *b* = *b*‐value; *S*_0_ = signal at the shortest echo time with diffusion weighting of zero; *f* = perfusion fraction; TE_min_ = shortest echo time acquired; T2*_fast_ and T2*_slow_ = the effective T2* associated with the perfusion and diffusion compartments respectively; D* = pseudo‐diffusion coefficient (associated with the perfusion compartment); ADC = apparent diffusion coefficient (associated with the diffusion compartment).

### 2.5 Outcome data

Outcome data including delivery outcomes such as gestation at birth, the onset of labour, mode of delivery, birthweight and birthweight centile (calculated via INTERGROWTH‐21)^26^ were collected. Neonatal outcomes including neonatal unit admission, need for ventilatory support, and severe neonatal morbidities such as intraventricular haemorrhage and necrotising enterocolitis were noted. Maternal complications including severe sepsis were also collected. Placental histopathology was determined for all women who delivered preterm: all lesions were noted, with chorioamnionitis reported as per the Amsterdam criteria.^10^

### 2.6 Statistical analysis

As this is a pilot study exploring a novel hypothesis and technique no power calculation was undertaken. Demographic and neonatal outcome data were analysed using Student's *t*‐test for continuous data and a Chi‐squared test for categorical data. After testing for normality, linear regression was used to test for difference between groups, including a subgroup analysis of the preterm cases considering whether membranes were intact. For cases, a ratio was created [gestation at onset of symptoms to gestation at MRI] to [gestation at delivery to gestation at MRI], with decreasing scores representing a closer time to delivery than the first symptom (diagnosis of PPROM or bulging membranes) and data transformed to standardised variables. For analysis, women were split into binary groups of scores above and below zero as zero represents the midpoint of the now normally distributed data. All analyses were undertaken in SPSS version 29 and graphs were made in Excel version 16.75. Significance was set at *p* < 0.05. Results are reported in line with the STROBE statement which can be found in Table S1.^27^

## 3 RESULTS

### 3.1 Recruitment of participants

Twenty‐three women who subsequently delivered prior to 32 weeks' gestation were included in the analysis (Figure S1). Of these, 14 had PPROM at the time of the scan. Seven were on prophylactic oral antibiotics at the time of MRI, all of whom had PPROM. Mean time from MRI to delivery was 8 days (SD 8.7). Placental histopathology was available for 19 cases: 14 demonstrated chorioamnionitis with 12 also having funisitis, and none had any other anomalies noted. A total of 52 controls, all of whom delivered at term and without any maternal or fetal complications were included. Table S2 summarises the characteristics of all participants.

### 3.2 Single compartment models

**FIGURE 1:**
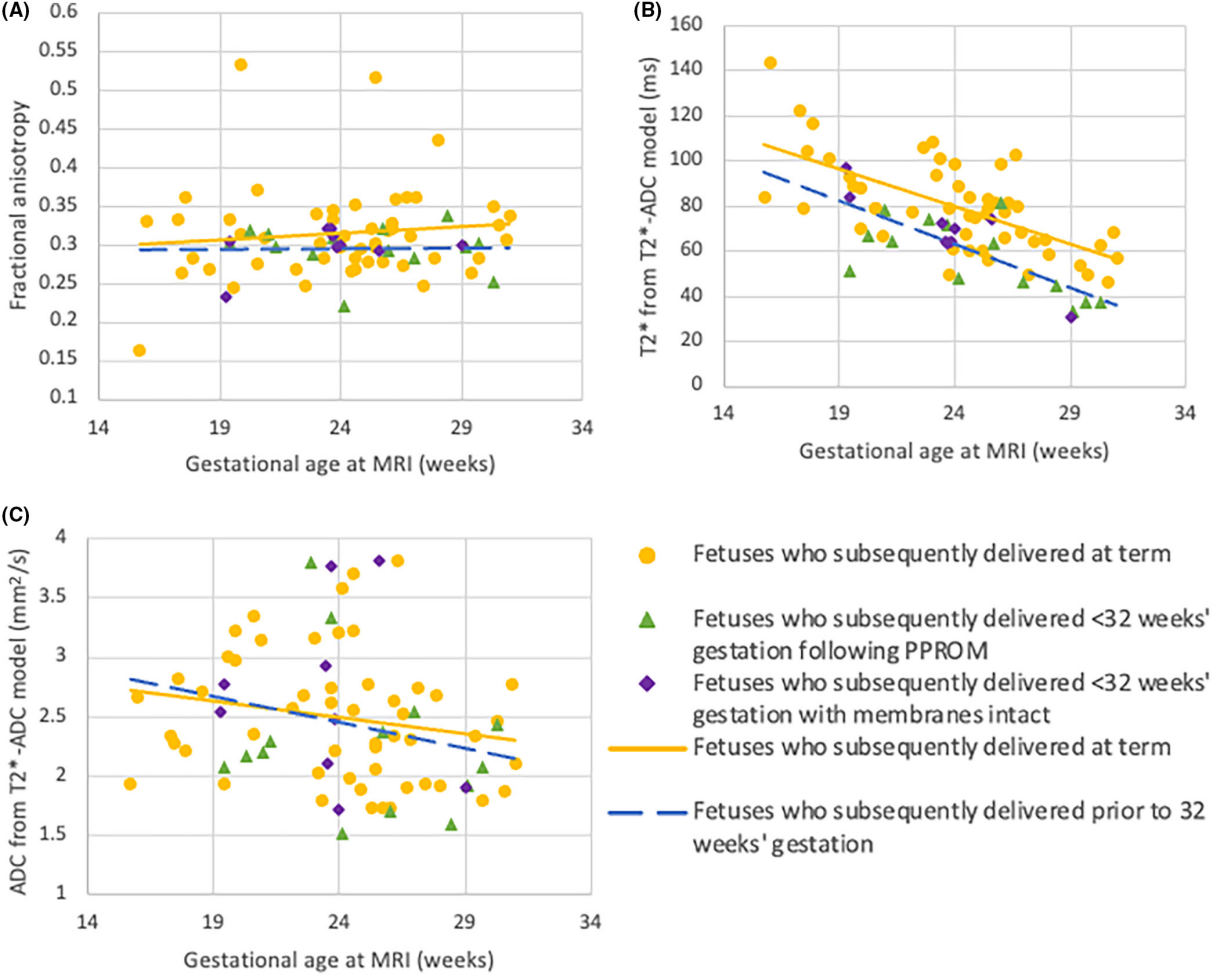
Fractional anisotropy and T2*‐ADC model in foetuses who subsequently deliver <32 weeks as compared to those who deliver at term. *Statistically significant result. (A) Fractional anisotropy (*p* = 0.746). (B) T2 (*p* < 0.001); (C) ADC (*p* = 0.767). Yellow circles: Foetuses born at term; green triangles: Foetuses who deliver <32 weeks following PPROM; purple diamonds: Foetuses who deliver <32 weeks' with membranes intact; yellow, solid line: Foetuses who deliver at term; blue, dashed line: Foetuses who subsequently deliver <32 weeks.

Fractional anisotropy was consistent across the gestational range and was unaffected by subsequent preterm birth (*p* = 0.087 and *p* = 0.746, respectively). Across gestation, there was a significant downward trend in T2* but not ADC among the controls (*p* < 0.001, and *p* = 0.163, respectively). The T2* was significantly reduced in the cohort who subsequently delivered preterm (*p* < 0.001), and this was more significant in the PPROM group than the membranes intact group (*p* < 0.001 and *p* = 0.027, respectively). The ADC was not significantly different in the cohort who subsequently delivered preterm (*p* = 0.767). Findings are summarised in Figure 1.

### 3.3 T2*‐IVIM model

**FIGURE 2:**
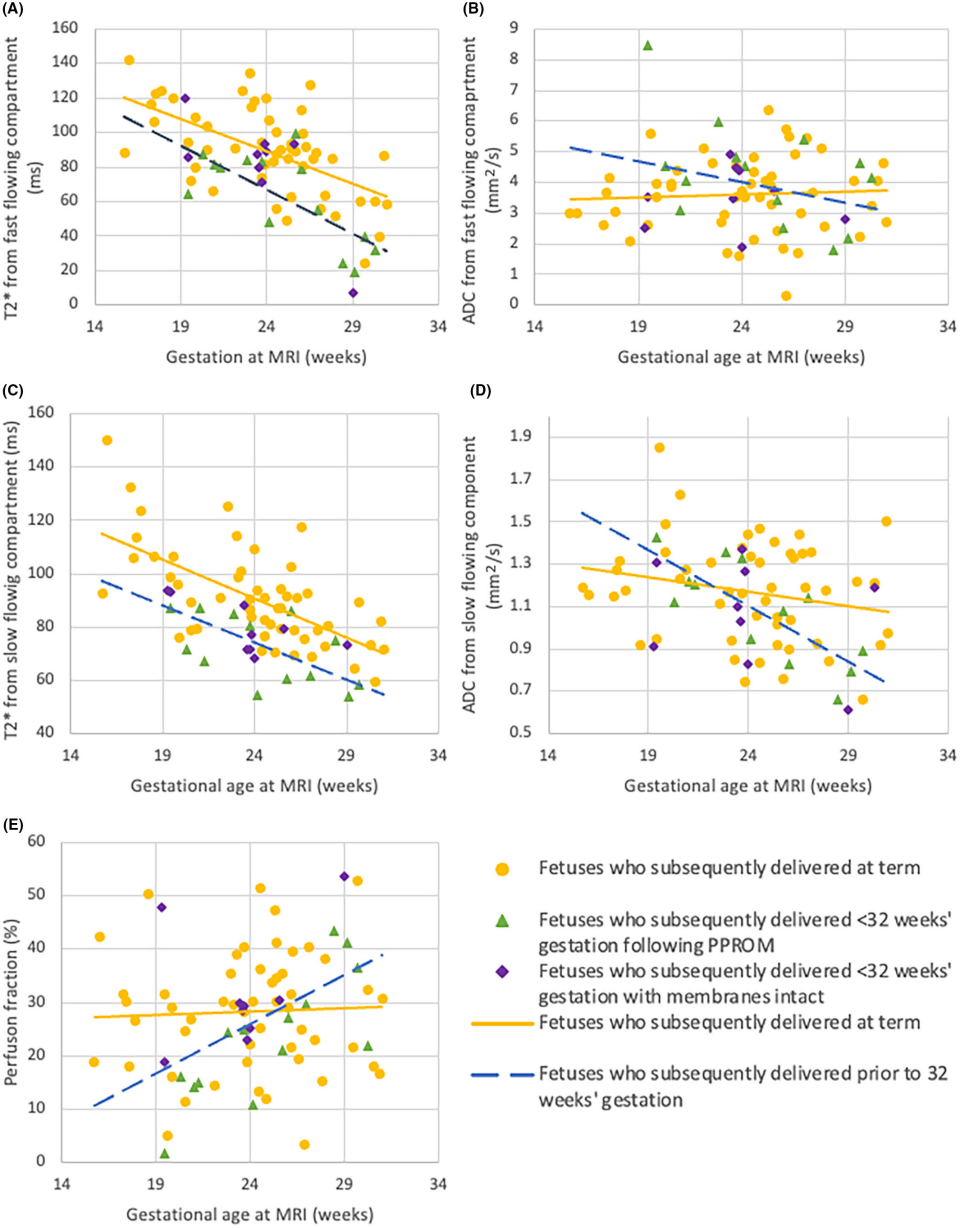
T2*‐IVIM model in foetuses who subsequently deliver <32 weeks as compared to those who deliver at term. (A) T2* in the fast‐flowing component (*p* = 0.001); (B) ADC in the fast‐flowing component (*p* = 0.688); (C) T2* from slow‐flowing component (*p* < 0.001); (D) ADC from the slow‐flowing compartment (*p* = 0.177); (E) Perfusion faction (*p* = 0.546). Yellow circles: Foetuses born at term; green triangles: Foetuses who deliver <32 weeks following PPROM; purple diamonds: Foetuses who deliver <32 weeks' with membranes intact; yellow, solid line: Foetuses who deliver at term; blue, dashed line: Foetuses who subsequently deliver <32 weeks.

T2* values decreased across gestation in both the fast and slow compartments (*p* < 0.001 for both). A decrease in T2* was observed in the fast‐flowing compartment in foetuses who subsequently delivered preterm (*p* = 0.001). This was driven by the PPROM group (*p* < 0.001 in the PPROM group vs. *p* = 0.182 in the membrane intact group). In the slow‐flowing compartment, there was a decrease in T2* (*p* < 0.001) with significant differences seen in both the PPROM and membrane intact groups (*p* < 0.001 and *p* = 0.013, respectively). The ADC was not significantly impacted by gestation in either compartment among the controls (*p* = 0.688 and *p* = 0.155 for fast‐ and slow‐flowing, respectively), and no difference was noted in either compartment among foetuses who subsequently delivered preterm (*p* = 0.255 and *p* = 0.177). The perfusion fraction was constant across gestations (*p* = 0.757) and unaffected by subsequent preterm birth (*p* = 0.546). Findings are summarised in Figure 2, and a summary of all findings in both models is given in Table S3.

### 3.4 Further subgroup analysis

**FIGURE 3:**
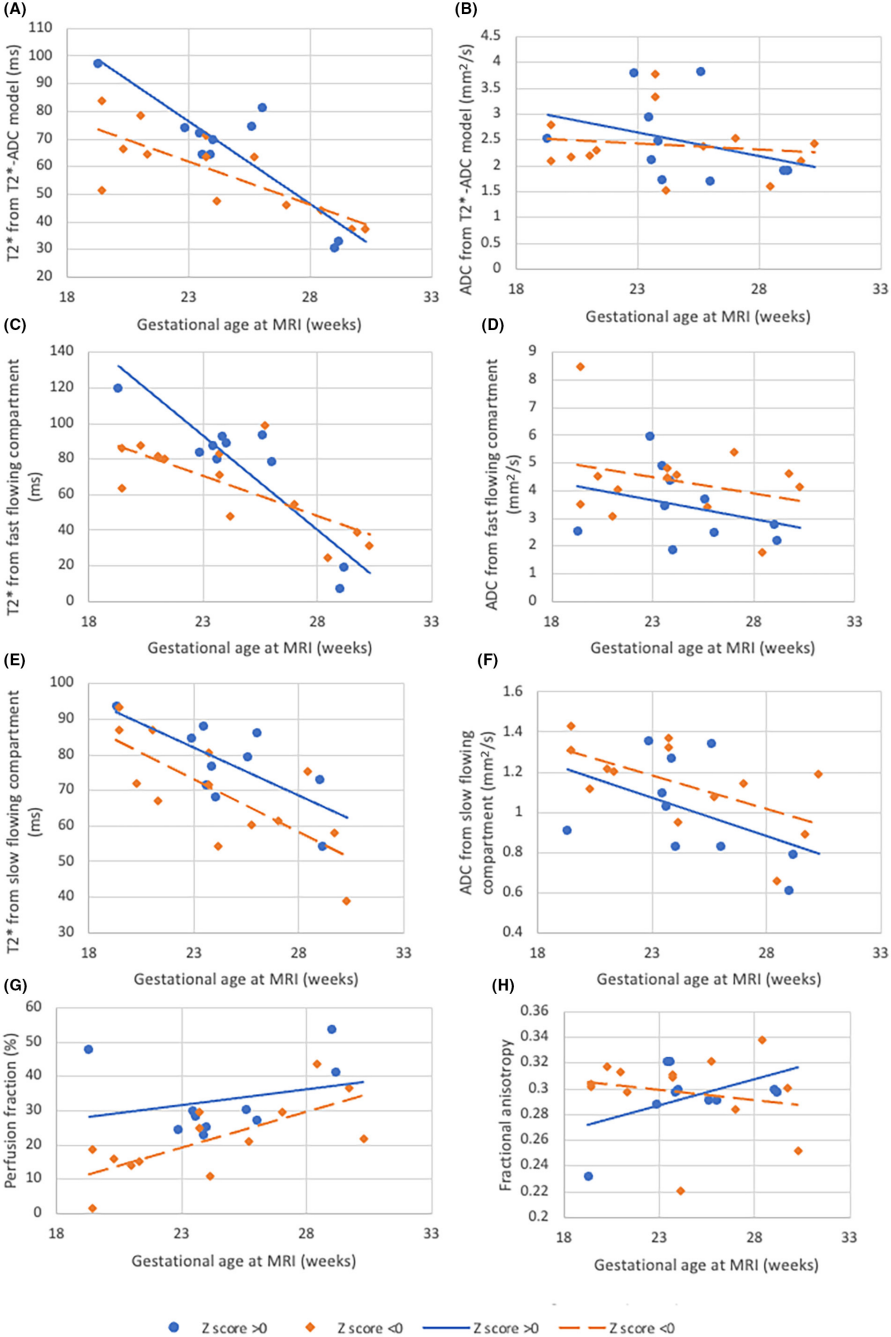
Ratio demonstrating latency from symptom onset to delivery for all high‐risk participants. A score of <0 indicates that a woman is further through the process from onset of symptoms to delivery. *Statistically significant result. (A) T2* from T2*‐ADC model (*p* = 0.041); (B) ADC from T2*‐ADC model (*p* = 0.693); (C) T2* from the fast‐flowing compartment (*p* = 0.121); (D) ADC from the fast‐flowing compartment (*p* = 0.158); (E) T2* from the slow‐flowing compartment (*p* = 0.033); (F) ADC from the slow‐flowing compartment (*p* = 0.177); (G) Perfusion fraction (*p* = 0.015); (H) fractional anisotropy (*p* = 0.069). Blue circle: >0; orange diamond: <0; blue, solid line: >0; orange, dashed line: <0.

Analysis by interval to delivery time among the cases was conducted: among foetuses with a score < 0, T2* from the T2*‐ADC model, T2* in the slow‐flowing compartment and the perfusion fraction were reduced as compared to those with a score > 0 (*p* = 0.041, *p* = 0.033, *p* = 0.015 (Figure 3)). Numbers of foetuses without chorioamnionitis were too small to be able to meaningfully account for histopathology, but results by histopathological findings are summarised in Figure S2.

## 4 DISCUSSION

### 4.1 Summary of findings

In this cohort of 23 women who delivered preterm and 52 controls T2* from the T2*‐ADC model was lower in the preterm cases, and this was true in both the fast‐ and slow‐flowing compartments. On subgroup analysis, these changes were driven more by the PPROM subgroup in both compartments. The intact membranes subgroup had no change in the fast‐flowing compartment, and this finding was also noted when comparing women relatively closer to delivery. This highlights the value of a model that discriminates between compartments as it allows for the interrogation of more subtle differences that could highlight differing points in disease progression.

### 4.2 Strengths and limitations

The bicompartmental technique described here is of value to the placenta owing to its unique structure, with both maternal and fetal blood circulating. This allows for biological conjecture that the fast‐flowing compartment represents perfusing blood from the maternal circulation via the spiral arteries, with some fetal contribution as this enters the placental circulation; with the slow‐flowing compartment representing diffusing blood in microvilli and the intervillous spaces.^28^ Table 1 summarises the clinical interpretation of the techniques utilised. This is the first study to utilise diffusion‐relaxation techniques to interrogate placental changes prior to preterm birth in women with and without PPROM. Despite the technical challenges associated with scanning women at imminent risk of preterm birth this study has achieved sufficient pilot data to demonstrate clear deviations from normality. The scan described here is designed specifically for placental analysis, can be achieved in under 10 min and all scanning and postprocessing techniques are freely available. A major confounder in all studies examining antenatal antecedence to preterm birth is the variable time from presentation to delivery as it is likely that findings are related to this temporal relationship; we have accounted for this statistically.

**TABLE 1:** Summary of clinical interpretation of techniques used (example images from one control fetus scanned at 23 + 5 weeks' gestation, and one with PPROM scanned at 24 + 1 weeks' gestation).

Parameter	Clinical interpretation	Control example (23 + 5 weeks' gestation)	Case example (24 + 1 weeks' gestation, PPROM)
Fractional anisotropy	Extent of directional restriction of diffusion in placental tissue	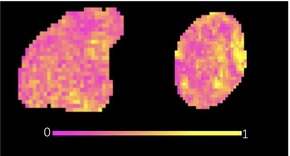	
T2* from T2‐ADC	Proxy marker of tissue oxygenation across the whole placenta	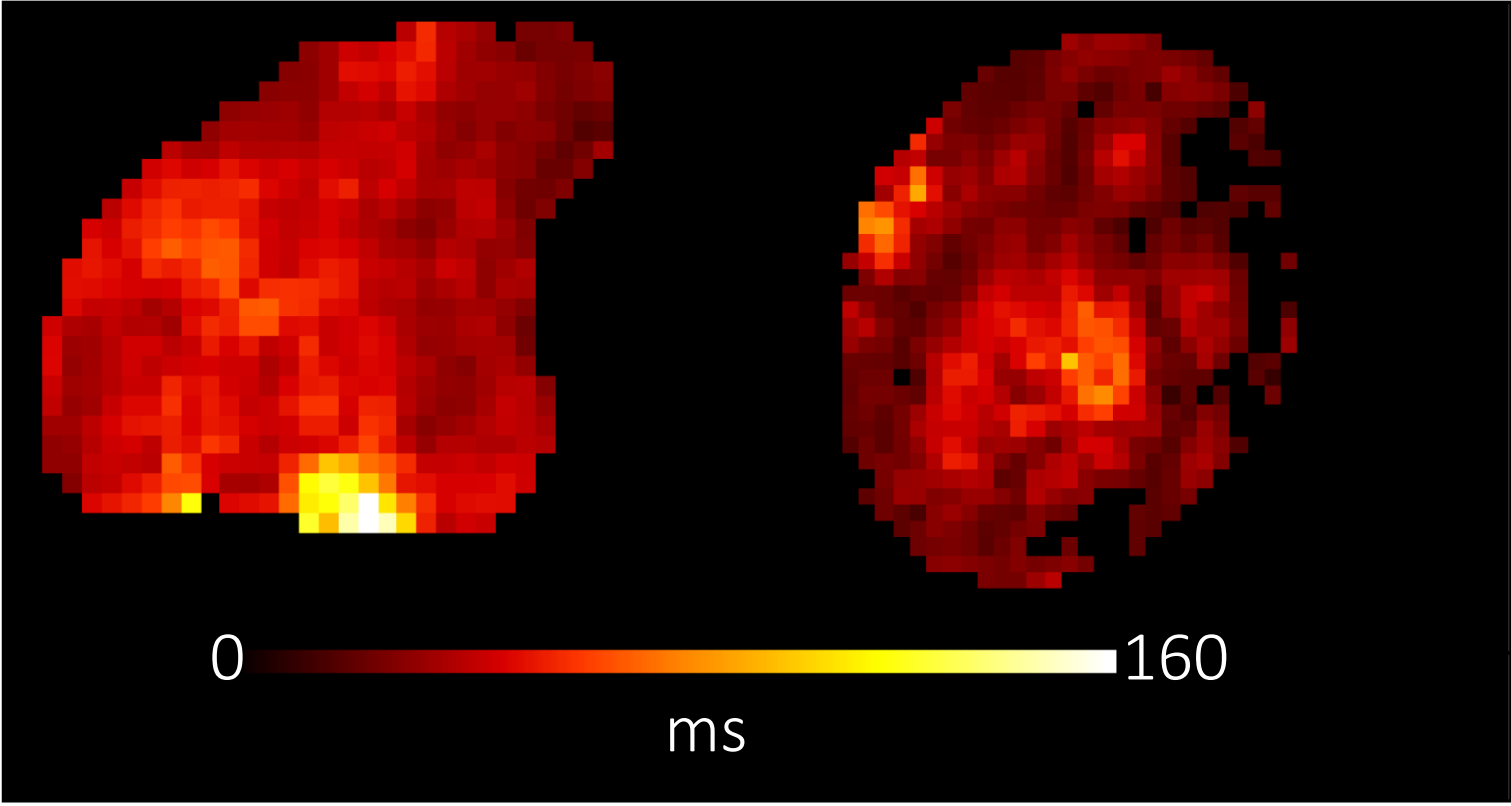	
ADC from T2*‐ADC	Complexity of tissue microstructure across the whole placenta	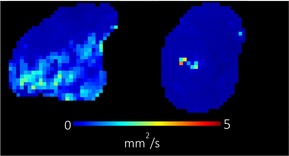	
Fast‐flowing T2*	Proxy marker of tissue oxygenation in the parts of the placenta fast‐flowingving fast‐flowing, perfusing, blood (eg from the maternal spiral arteries and large fetal vessels)	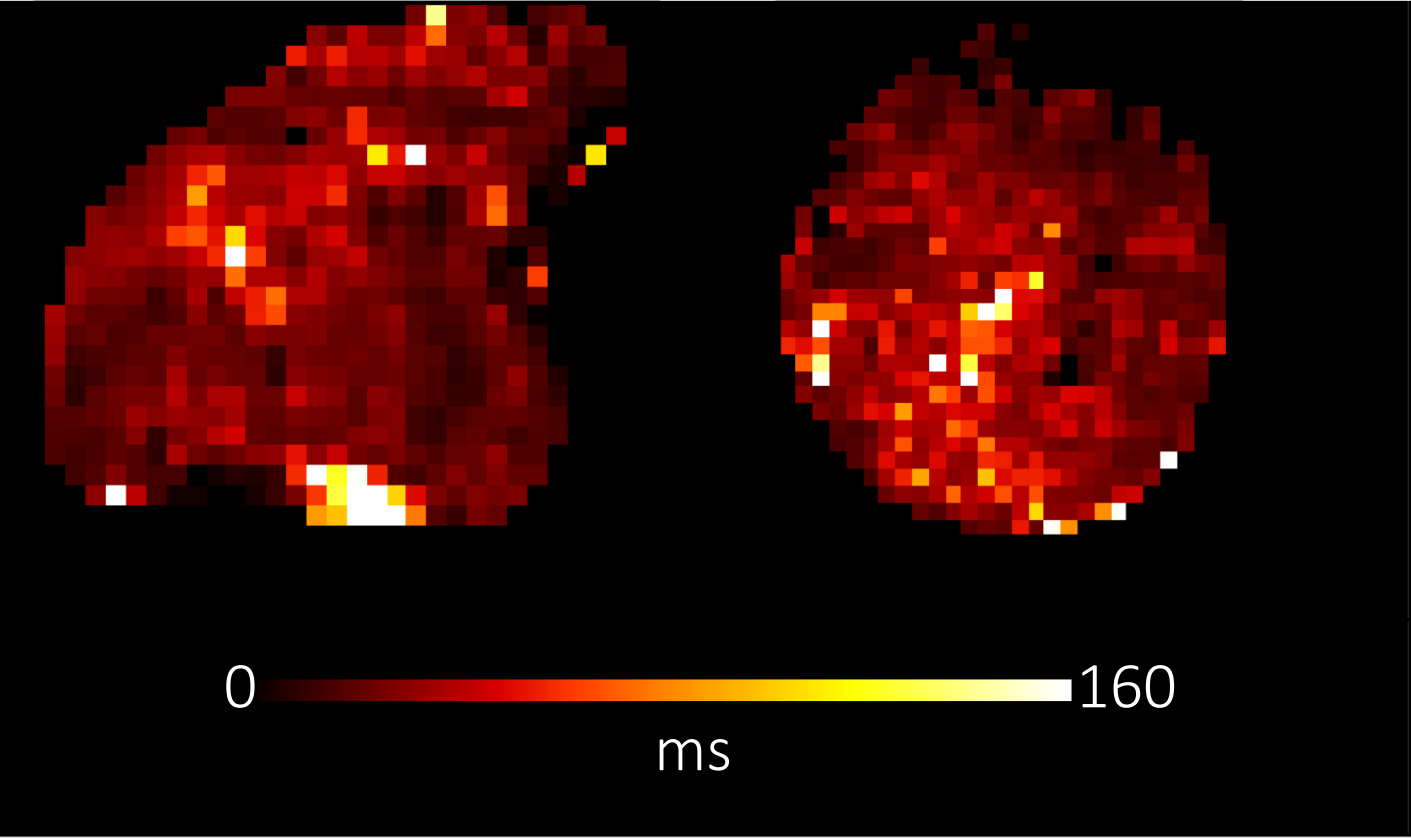	
Fast‐flowing ADC	Complexity of tissue microstructure in the same region as fast‐flowing T2*	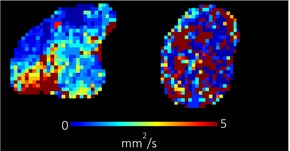	
Slow‐flowing T2*	Proxy marker of tissue oxygenation in the parts of the placenta that receive slow‐flowing, diffusing blood (eg. microvilli and intervillous spaces)	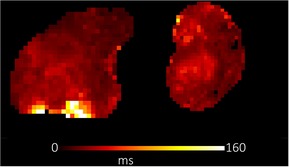	
Slow‐flowing ADC	Complexity of tissue microstructure in the same regions as slow‐flowing T2*	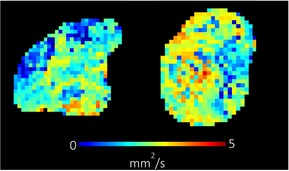	
Perfusion fraction	Proportion of each voxel where the fast‐flowing fraction predominates	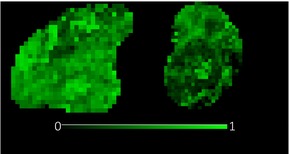	

Abbreviation: ADC, apparent diffusion coefficient.

However, owing to small numbers we could only analyse this data in binary groups; it is very likely that the microstructure of the placenta will change from onset of PPROM or bulging membranes to delivery, and further interrogation via a larger sample size is warranted. This would also allow for subgroup analysis based on histopathology and could overcome the imbalance in ethnic backgrounds of women participating thus far. The methods described here are highly sensitive to the region of interest: this study was restricted to the placental parenchyma. Additional areas of interest may include the basal plate or a more restricted segmentation of the placental periphery given ascending vaginal infection seems to progress from the periphery towards the centre of the placenta.^29^ Interrogation of these regions may add further detail to our understanding of chorioamnionitis in vivo.

### 4.3 Interpretation

While there is consistent evidence of reducing mean placental T2* across gestation,^28^, ^30^ findings related to placental ADC and perfusion vary. Some studies support an increase in perfusion fraction over gestation,^14^, ^17^ but there is seemingly contradictory evidence for a decrease in placental perfusion, or alternatively a stable relationship.^31^, ^32^ This is intriguing and while the cause is not clear, one study investigating the in‐participant rate of change of placental perfusion does highlight that in‐participant change was not consistent, suggesting that larger datasets may be required for true normal trends to be noted.^14^ Previous work from our group also suggests a significant reduction in ADC over gestation relies on data to term and changes later in gestation than included here may drive these significant changes.^17^ Given that maternal blood flow to the uterine arteries does not increase in a linear fashion, and that the placenta undergoes normal histological changes throughout gestation, larger datasets may reveal more regarding normal trends.^33^

Our previous work looking at the PPROM group has demonstrated similar findings to those described here.^17^, ^34^ Inclusion of the group with membranes intact has given some clarity on the relative impact of both clinical situations: while both result in preterm birth, there are slightly more severe (but overlapping) changes noted in the PPROM group, perhaps reflecting the higher rate of chorioamnionitis and funisitis diagnosed in this group. The differences reported between the control population and both subgroups of the preterm birth population are as anticipated given the histological findings of chorioamnionitis, and the pathophysiology of the fetal inflammatory response syndrome. The influx of neutrophils and eosinophils, and necrotic processes would be anticipated to result in the faster T2* decay noted here; the increased decay on the fetal side may represent equivalent changes secondary to fetal inflammatory response syndrome. While FA and ADC results are not significantly different, trends across gestation are dissimilar, perhaps pointing towards microstructural changes that could be further elucidated with larger samples that could be subgroup analysed across smaller gestational ranges. The reduction in perfusion fraction noted in the subgroup of women relatively closer to delivery is novel and may represent necrotic processes prior to the onset of labour, although this would have to be interrogated further. While it is not possible to elucidate these differences in the current study, all placental findings prior to preterm birth should be considered in light of maternal vascular lesions and their association with preterm birth in women without chorioamnionitis.^4^, ^35^

While this study points towards the ability of MRI to provide clinically accurate risk prediction for preterm chorioamnionitis, a larger sample size would be needed first to confirm findings, and then link to clinical outcome. Placental MRI is an active area of research and, as such, continuous improvement in acquisition techniques is likely to result in greater post‐processing potential, including more regional analysis, which is likely to be of further benefit to this work. While expedited delivery is the only management of chorioamnionitis at any gestation, it is most likely that clinical utility of this technique would be related to uncertain diagnosis of chorioamnionitis at earlier viable gestations where the consequences of both a false positive and a false negative diagnosis are severe. While chorioamnionitis is the prevailing histopathological diagnosis at this gestation, it should be noted that maternal vascular malperfusion lesions are also more common in this group. Previous work utilising diffusion techniques in relation to pre‐eclampsia would suggest that the patterns of abnormalities seen here would be different to those seen in chorioamnionitis, and this should be confirmed in a direct comparison between the two groups. Application of more complex postprocessing techniques, such as machine driven analysis^36^ may add granularity to our understanding of the pathophysiological mechanisms underlying placental disease, but clinical considerations for their use must include ease of application and interpretation alongside current clinical investigations such as maternal biochemistry and ultrasound derived cervical length.

Although this study highlights the potential for MRI to provide clinically meaningful data on the placenta prior to preterm birth, it should be recognised that ultrasound remains a more widely available and cheaper technique, both in terms of access to scanning and in expertise in interpretation among obstetricians. While B‐mode has not proven to be of clinical value in the prediction of chorioamnionitis, there is potential for relevant interrogation of placental function via placental power Doppler and the use of microbubble contrast agents: both techniques have been demonstrated to give information on placental perfusion. Microbubble contrast agents have not been utilised outside of the first trimester in humans and safety has not been fully established.^37^

## 5 CONCLUSION

While accurate antenatal prediction of preterm chorioamnionitis remains elusive, these findings support the further investigation of advanced MRI techniques for in vivo assessment of the placenta. However, the small sample size here and the need for greater discrimination between placenta findings in preterm birth as compared to alternative or concurrent obstetric diseases, as well as the impact on maternal and neonatal outcomes must be considered and further investigated. However, in the future, MRI may have application in determining safest time to deliver women suspected of having preterm chorioamnionitis.

## AUTHOR CONTRIBUTIONS

MH methodology, formal analysis, investigation, data curation, writing – original draft, writing – review and editing, visualisation; NS: writing – review and editing, supervision; MR: writing – review and editing; AS writing – review and editing, supervision; JH conceptualisation; methodology, software, writing – review and editing, funding acquisition; LS conceptualisation; methodology; investigation; resources; data curation; writing – review and edition; supervision; funding acquisition.

## FUNDING INFORMATION

LS is funded by the National Institute for Health and Care Research (NIHR) (NIHR Advanced Fellowship [301664]). This work was supported by core funding from the Wellcome/EPSRC Centre for Medical Engineering [WT203148/Z/16/Z], by the National Institute of Health (NIH) Human Placenta Project grant 1U01HD087202‐01 (Placenta Imaging Project (PIP)), by the Wellcome Trust, Sir Henry Wellcome Fellowship to JH, [201374/Z/16/Z], by the UKRI, FLF to JH [MR/T018119/1] and by the National Institute for Health and care Research (NIHR) Biomedical Research Centre based at Guy's and St Thomas' NHS Foundation Trust and King's College London.

## CONFLICT OF INTEREST STATEMENT

The authors report no conflicts of interest.

## ETHICS APPROVAL

Data were acquired as part of the Individualised risk prediction of adverse neonatal outcomes in pregnancies that deliver preterm using advanced MRI techniques and machine learning, ethical approval code 21/SS/0082 and The use of advanced MRI techniques to evaluate antenatal lung development, ethical approval code 22/YH/0210. Additional retrospective data were taken from Antenatal assessment of fetal infection using advanced MRI protocols, ethical approval code 19/SS/0032; and from The placental imaging project, ethical approval code 16/LO/1573; The congenital heart imaging programme, ethical approval code 21/WA/0075. All women gave written consent prior to participation.

## Supporting information


Figure S1.

Figure S2.

Table S1.

Table S2.

Table S3.

## Data Availability

The dataset generated and analysed for this study is available upon reasonable request to the authors.
