# Supplementary figures and images for: Placental multimodal MRI prior to spontaneous preterm birth <32 weeks' gestation: An observational study
Source: BJOG. 2024 Jul 2;131(13):1782–92. doi: 10.1111/1471-0528.17901 (PMC11801328; doi:10.1111/1471-0528.17901)

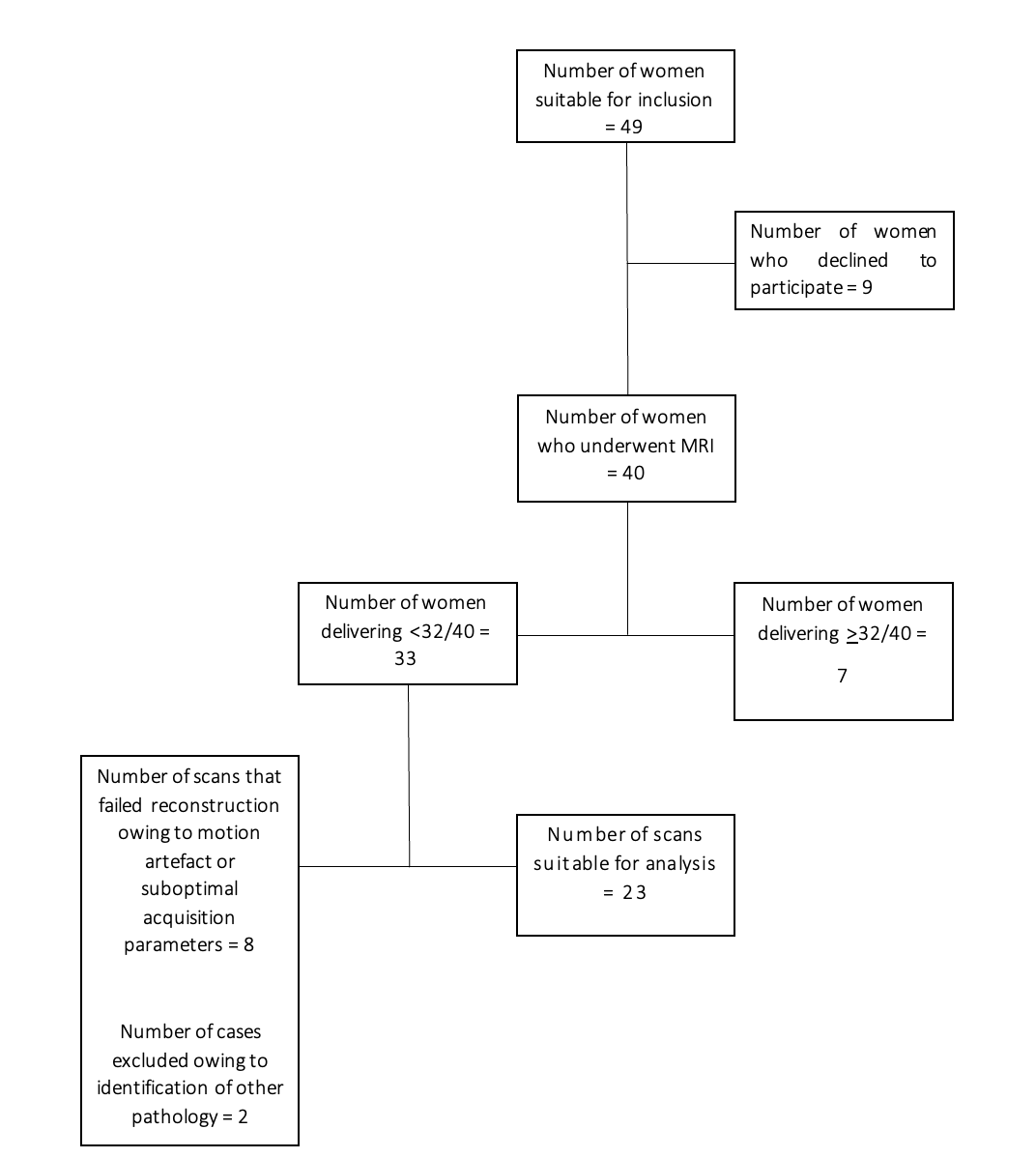

Supplement: Supplementary file 1 — Figure S1. [file BJO-131-1782-s002.png]

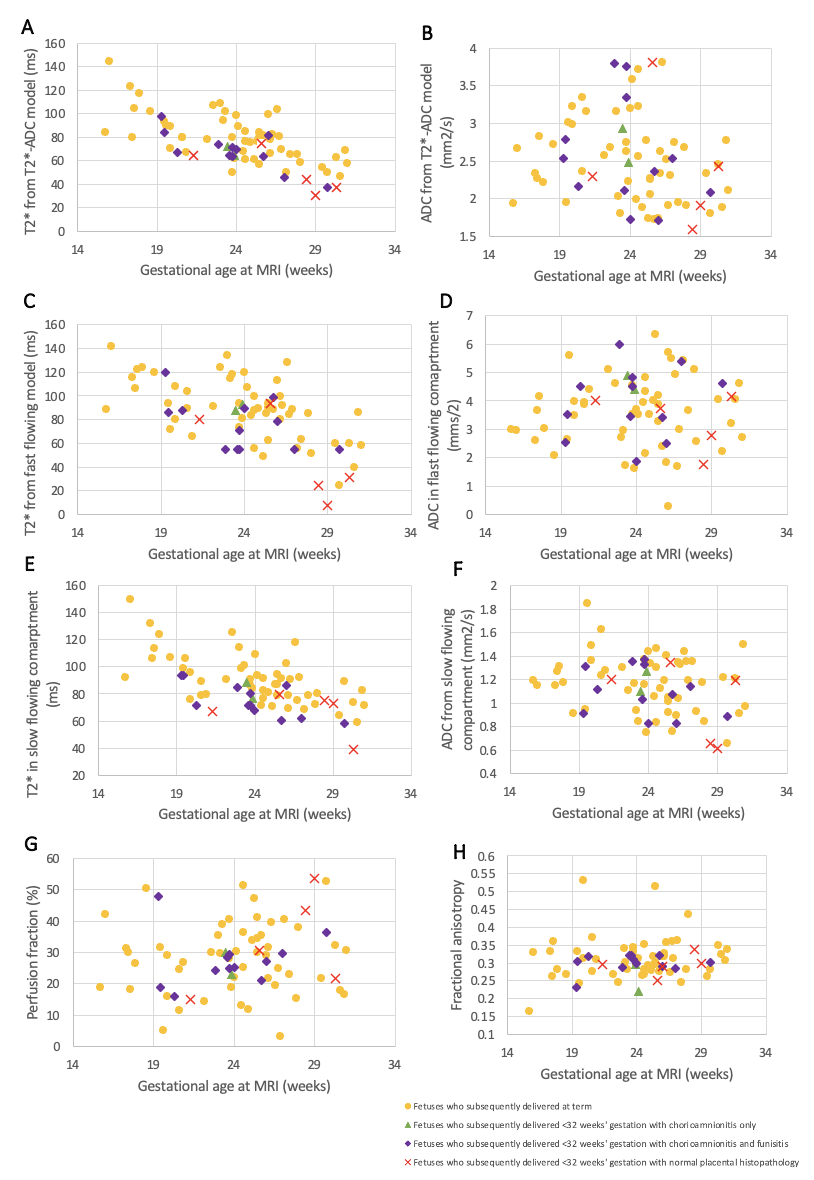

Supplement: Supplementary file 2 — Figure S2. [file BJO-131-1782-s004.tiff]
