# Supplementary material for: Placental multimodal MRI prior to spontaneous preterm birth <32 weeks' gestation: An observational study
Source: BJOG. 2024 Jul 2;131(13):1782–92. doi: 10.1111/1471-0528.17901 (PMC11801328; doi:10.1111/1471-0528.17901)
Supplement: Supplementary file 4 — Table S2. [file BJO-131-1782-s001.docx]

| **Characteristic** | **Term Cohort (n=52)** | **Preterm Cohort (n=23)** | **p** | **95% CI** |
| --- | --- | --- | --- | --- |
| **Maternal age (y)**  Mean (SD) | 34.9 (4.2) | 31.1 (3.9) | 0.041 | -  0.19 to 7.87 |
| **BMI (kg/m^2^)**  Mean (SD) | 23.5 (2.8) | 23.6 (3.3) | 0.899 | -1.74 to 1.83 |
| **Ethnicity, n (%)** |  |  |  |  |
| White | 45 (87) | 13 (57) |  |  |
| Black | 2 (4) | 4 (17) |  |  |
| South Asian | 2 (4) | 3 (13) |  |  |
| East Asian | 1 (1) | 0 |  |  |
| Other | 2 (4) | 3 (13) |  |  |
| **Ethnicity grouping, n (%)** |  |  |  |  |
| White | 45 (87) | 13 (57) | **0.004** |  |
| Non-white | 7 (13) | 10 (43) |  |  |
| **Parity, n (%)** |  |  |  |  |
| Primiparous | 33 (63) | 13 (57) | 0.569 |  |
| Multiparous | 19 (37) | 10 (43) |  |  |
| **Gestation at MRI (wk)**  Mean (SD) | 23.9 (3.9) | 24.4 (3.4) | 0.657 | -2.2 to 1.39 |
| **Gestation at birth (wk)**  Mean (SD) | 40 (1.1) | 25.57 (3.6) | **<0.001** | 12.9 to 16.08 |
| **Birth weight (g)**  Mean (SD) | 3390.2 (591.03) | 879.5 (536.4) | **<0.001** | 2226.9 to 2794.3 |
| **Birthweight centiles of live births, n (%)*** |  |  |  |  |
| 0-3 | 0 | 0 | 0.190 |  |
| 3-10 | 2 (4) | 3 (13) |  |  |
| 10-25 | 2 (4) | 0 |  |  |
| 25-50 | 10 (19) | 0 |  |  |
| 50-75 | 14 (27) | 7 (30) |  |  |
| 75-90 | 16 (31) | 4 (17) |  |  |
| 90-97 | 8 (15) | 2 (9) |  |  |
| 97-100 | 0 | 0 |  |  |
| **Sex of infant, n (%)** |  |  |  |  |
| Female | 32 (62) | 10 (43) | 0.146 |  |
| Male | 20 (38) | 13 (57) |  |  |
| Undetermined | 0 | 0 |  |  |
| **Outcome, n (%)** |  |  |  |  |
| Live at discharge | 52 (100) | 13 (57) | **<0.001** |  |
| Death prior to discharge: | 0 | 10 (43) |  |  |
| *Second trimester pregnancy loss* |  | *7 (30)* |  |  |
| *Neonatal death* |  | *3 (13)* |  |  |

*Continuous data analysed using Student's t-test; categorical data using Chi^2^.
BMI: body mass index; MRI: magnetic resonance imaging.
* Birthweight centiles are not available prior to 23 weeks’ gestation.*
