# Supplementary material for: Placental multimodal MRI prior to spontaneous preterm birth <32 weeks' gestation: An observational study
Source: BJOG. 2024 Jul 2;131(13):1782–92. doi: 10.1111/1471-0528.17901 (PMC11801328; doi:10.1111/1471-0528.17901)
Supplement: Supplementary file 5 — Table S3. [file BJO-131-1782-s003.docx]

|  | **Control slope p value, R^2^ value** | **Preterm birth p value, R^2^ value** | **PPROM p value, R^2^ value** | **Membranes intact p value, R^2^ value** |
| --- | --- | --- | --- | --- |
| **Fractional anisotropy** | 0.087, 0.059 | 0.760, 0.009 | 0.250, 0.034 | 0.096, 0.024 |
| **T2* from T2*-ADC** | **<0.001, 0.426** | **<0.001, 0.528** | **<0.001, 0.520** | **0.027, 0.457** |
| **ADC from T2*-ADC** | 0.163, 0.039 | 0.767, 0.041 | 0.289, 0.031 | 0.423, 0.045 |
| **Fast flowing T2*** | **<0.001, 0.338** | **0.001, 0.438** | **<0.001, 0.451** | 0.182, 0.345 |
| **Fast flowing ADC** | 0.688, -0.017 | 0.255, -0.007 | 0.099, 0.015 | 0.880, -0.031 |
| **Slow flowing T2*** | **<0.001, 0.405** | **<0.001, 0.512** | **<0.001, 0.515** | **0.013, 0.418** |
| **Slow flowing ADC** | 0.115, 0.049 | 0.177, 0.116 | 0.317, 0.081 | 0.292, 0.038 |
| **Perfusion fraction** | 0.757, -0.018 | 0.546, 0.009 | 0.121, 0.036 | 0.369, -0.015 |
